# Enhancing rare disease guideline development with real-world data: a method evaluation

**DOI:** 10.1186/s12874-026-02802-7

**Published:** 2026-02-14

**Authors:** Willemijn Irvine, Linde Margriet van der Kamp, Olivia Spivack, Alexandra Benachi, Carmen Mesas Burgos, Roel Bakx, Charlotte Gaasterland, Rene Wijnen

**Affiliations:** 1 Department of Pediatric Surgery, Erasmus MC Sophia Children’s Hospital, Rotterdam, the Netherlands; 2 Department of Evidence Based Medicine and Methodology, Qualicura Healthcare Support Agency, Breda, the Netherlands; 3 Dutch Institute for Clinical Auditing, Leiden, the Netherlands; 4 Department of Obstetrics and Gynecology, Antoine Béclère Hospital, Paris Saclay University, Clamart, France; 5 Department of Pediatric Surgery, Karolinska University Hospital, Stockholm, Sweden; 6 Department of Pediatric Surgery, Emma Children’s Hospital, Amsterdam UMC, Amsterdam, the Netherlands; 7 Department of Clinical Epidemiology, Leiden University Medical Center, Leiden, the Netherlands

**Keywords:** Rare diseases, Guideline, Real-world data, Methodology

## Abstract

**Background:**

Real-world data collection can contribute to quality improvement for rare diseases in several ways.

**Objectives:**

To evaluate a method developed to integrate real-world evidence in guidelines addressing rare diseases (REGARD).

**Methods:**

A guideline on omphalocele was developed following the GIN-McMaster Guidelines 2.0 checklist. After the guideline panel selected clinical questions, systematic literature searches were performed. When published evidence was scarce, REGARD was introduced. Data from the European Pediatric Surgical Audit (EPSA) patient registry was provided to clinical experts after which they noted their observations of treatment effect in structured observation forms (SOFs). Feasibility, added value and acceptability of REGARD were evaluated using a cross-sectional study design.

**Results:**

EPSA data were available for seven of the twelve clinical questions selected for this guideline (58%). In 4/7 clinical questions (57%), REGARD contributed to the panels’ decision on a recommendation. Reasons to disregard the supplementary evidence during the formation of a recommendation were: uncertainty about the validity of the observations due to small sample sizes and doubt about the association between the observed outcomes and the intervention because the registry data lacked information on the clinical indication for a chosen treatment.

**Conclusions:**

This study evaluates a method to connect real-world data collection to guideline development in rare diseases, highlighting both the challenges and benefits this method has to offer. Although the methodology requires refinement and the EPSA registry must be optimized, we believe it holds significant promise as a tool for developing guidelines for rare diseases.

**Supplementary Information:**

The online version contains supplementary material available at 10.1186/s12874-026-02802-7.

## Background

**Fig. 1:**
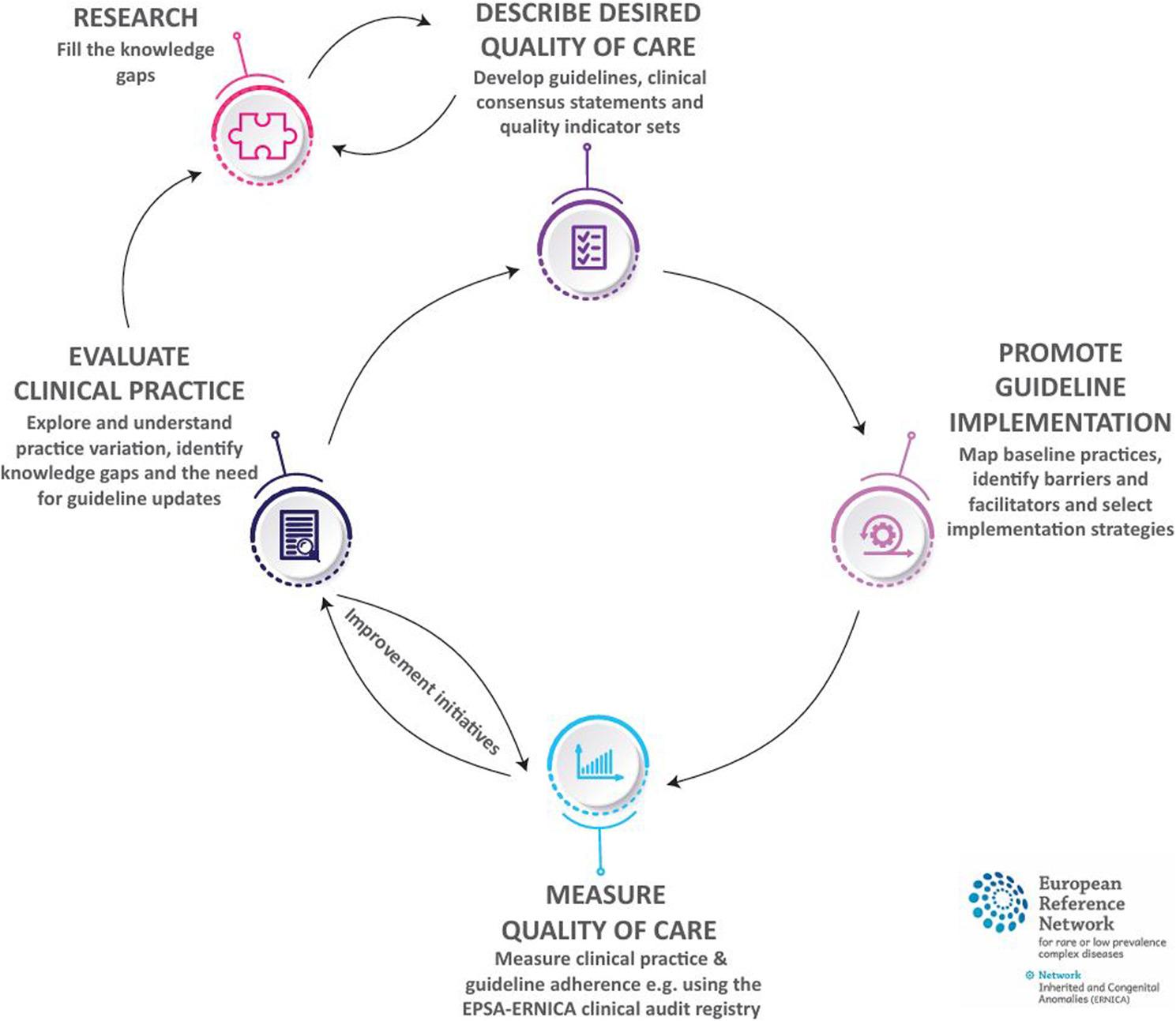
The ERNICA quality cycle. Adopted from Spivack et al. [2]

The European Reference Network for Rare Inherited Congenital Anomalies (ERNICA) is a network dedicated to improving quality of care for patients with rare digestive and gastrointestinal diseases [1]. So far, clinicians and researchers from 52 expert hospitals in Europe form this network together with patient representatives, representing national and/or international patient organizations. ERNICA works on improving quality of care through an iterative quality improvement system, see Fig. 1.

To facilitate the measurement of care quality, ERNICA relies on the European Pediatric Surgical Audit (EPSA) [3]. The EPSA is a clinical audit registry in which real-world data is collected. In the context of the EPSA, *real-world data* are prospectively and systematically collected, capturing information on patient characteristics, treatments, and outcomes as they occur in routine clinical practice. Analysis of patient data within the EPSA enables benchmarking and identification of best practices across participating centers, since all centers contribute to the same standardized database. The EPSA is open to participate not only to ERNICA member hospitals, but to a wider group of European hospitals. Data collection started in 2014 in the Netherlands; today, 36 hospitals from 21 European countries are connected to the EPSA. Data is collected on the following rare conditions: esophageal atresia, Hirschsprung’s disease, necrotizing enterocolitis, congenital diaphragmatic hernia, malrotation, gastroschisis and omphalocele. The data dictionary for all registries, including all collected variables for each disease, can be downloaded from the website of data manager Medical Record Data Management (MRDM) [4].

Registry data from a clinical audit such as EPSA can serve as a valuable resource for quality improvement at various stages of the quality cycle. First, it could be used to assess whether there is clinical practice variation. Identifying variations in clinical practice could indicate the need to clearly define the desired level of care, either by creating new guidelines or revising existing ones. Second, data collection provides insight into guideline adherence and implementation success after the introduction of new guidelines or after specific implementation efforts. Third, registry data could be employed as a source of supplementary evidence for the development of new guidelines when published evidence is scarce. If implemented properly, guidelines have the potential to reduce clinical practice variation and improve patient outcomes [5]. However, developing trustworthy guidelines for the treatment of rare diseases has been proven challenging due to the absence of high-quality scientific evidence and outcome heterogeneity [6, 7]. This is also the case for the rare congenital anomalies covered by ERNICA, which typically occur in less than 1 in 2000 live births in the European Union [8]. Consequently, the absence of high-quality evidence complicates the application of evidence-based methods for guideline development, potentially hindering the ability to draw conclusions and form recommendations. Currently, alternative approaches such as consensus statements and Delphi studies are commonly used. However, there is a need to further develop innovative strategies that can support the creation of evidence-based guidelines, particularly in rare diseases, as delays in generating robust evidence can hinder timely clinical decision-making and access to optimal care.

Supplementing published evidence with real-world data has the potential to help overcome the challenge of limited evidence in the development of guidelines for rare diseases. It is suggested that a robust registry can be seen as equivalent to a high-quality observational study, as registries provide real-world data by capturing patient information prospectively [9, 10]. Thereby, regulatory bodies such as the European Medicines Agency could accept registry based-studies as a valid source of evidence for regulatory decisions, if the supporting registry is of sufficient quality and the objectives, design and analytical plan of the proposed study align [10, 11]. The RARE-Best practices Working Group concluded that incorporating patient registry data as a supplement is feasible and has high potential for creating better rare-disease guidelines [9]. Nevertheless, there is limited experience and information available on how to incorporate this into the guideline development process.

We therefore developed REGARD (**R**eal-world **E**vidence for **G**uidelines **A**ddressing **R**are **D**iseases), an innovative method to integrate real-world data into rare disease guideline development. This study evaluates our first experience with REGARD. The method was piloted concurrently with the development of a guideline on omphalocele, a rare congenital abdominal wall defect that is known for its high morbidity, clinical practice variation and limited availability of published evidence [12, 13].

## Methods

We conducted a pilot with REGARD and used a cross-sectional study design to perform a first evaluation of this method. Our primary research question was whether REGARD is of added value for the development of a rare disease guideline. To answer this question, we assessed feasibility, acceptability by guideline panels and contributions of REGARD to the recommendations of a guideline. The pilot with REGARD was conducted during the development of a guideline on omphalocele.

### Guideline development methods

The omphalocele guideline was developed according to the GIN-McMaster Guidelines 2.0 checklist [14]. A complete description of the methodology is included in the supplementary material (S1). Prior to the start of the guideline development process, a prioritization exercise was carried out within the network to decide which clinical questions were to be included for this guideline. These questions were formulated in the Patient Intervention Comparison Outcome (PICO) framework. The international guideline development panel consisted of five maternal and fetal medicine specialists, six neonatologists, twelve pediatric surgeons, a methodologist, an EPSA project manager, ERNICA project manager and an ERNICA implementation coordinator. After evaluation of the available evidence, the panel used the evidence to decision framework to reach agreement on recommendations [15]. During a peer review phase, external experts were invited to review the guideline before it was submitted.

### REGARD: integrating real-world evidence in guidelines addressing rare diseases

**Fig. 2:**
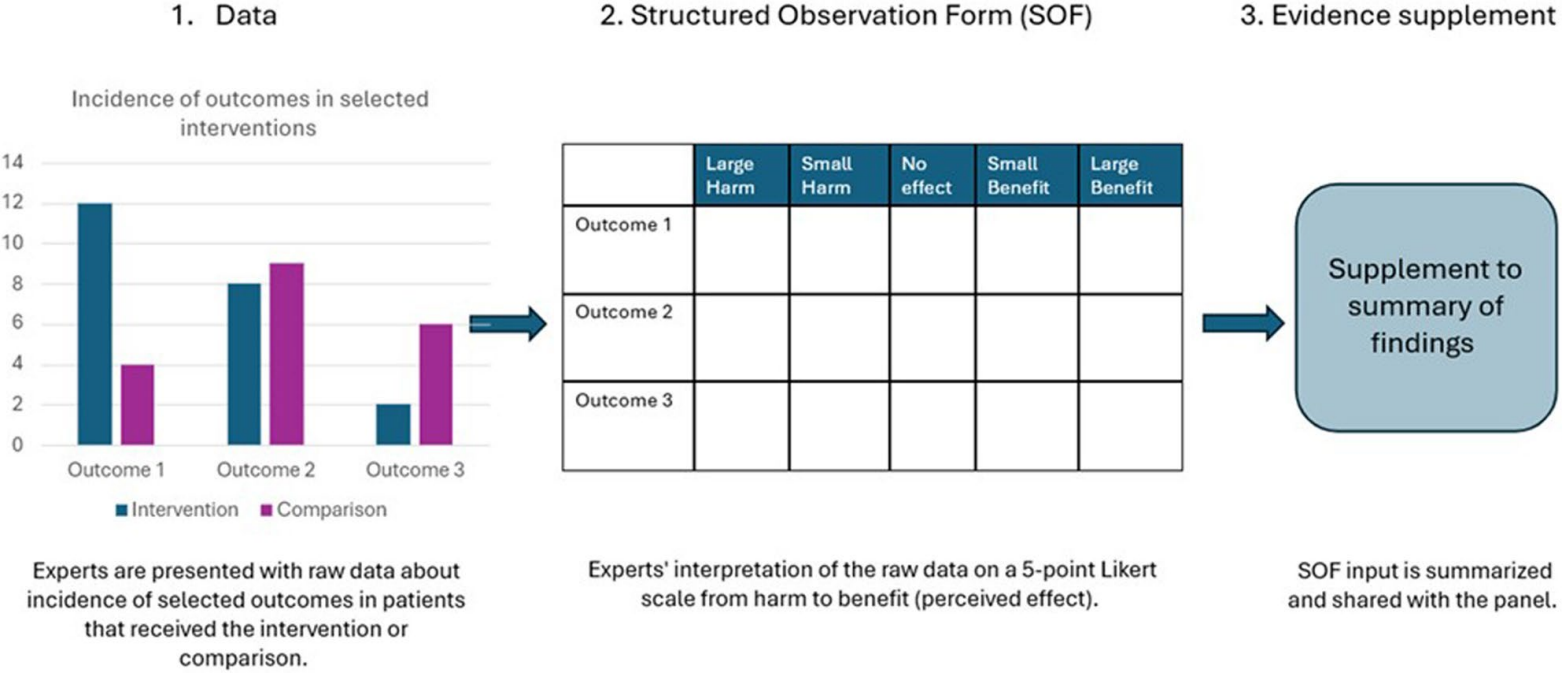
The process of REGARD: integrating registry data in guideline development through structured observation forms (SOFs)

With REGARD registry data is integrated in guideline development by using structured observation forms (SOFs). A SOF is a matrix that experts use to rate their perceived effectiveness of an intervention on selected outcomes on a spectrum from harmful to beneficial using a Likert type scale (see Fig. 2). Pai et al. [9] first suggested SOFs to structure expert opinion and allow panel members to describe observed effects in their own patient cases. For REGARD, we adopted similar principles but asked experts to evaluate EPSA data instead of their own patient cases using SOFs. The interventions and patient groups for which panel members received data to rate, were embedded in the clinical questions selected for the guideline. Completed SOFs were used as additional information during the guideline’s evidence to decision phase.

After a formal request, the Dutch Institute for Clinical Auditing (DICA) provided us with the raw data of all registered omphalocele patients in the EPSA between 2013 and 2024. In accordance with Dutch legislation, informed consent and ethical approval are not required, as the data is handled in an anonymized manner and the collection serves exclusively for quality improvement purposes. Additionally, our study protocol received approval from the EPSA Scientific Committee, that includes ethical considerations in their decision.

The EPSA data is prospectively collected from routine clinical practice and includes observational data. We used all available data without making randomized samples. According to current legal regulations, the data may be used internally for quality improvement, for example the data may be reviewed, analyzed and shared within the network, but cannot be published. To ensure that each clinical question could be appropriately addressed, available EPSA data variables were mapped against the elements of the PICO questions. Only questions for which the dataset contained sufficient variables to represent the population, intervention, comparison, and outcome were considered an adequate match and selected for inclusion in the pilot with REGARD. At the time of data extraction, the EPSA database contained data on 251 omphalocele patients registered from 2013 till 2023 [3]. Data collected between 2013 and 2020 represented only Dutch patients. From 2020 onwards, EPSA expanded internationally and data from patients in other European countries were included. Although there were data on 251 omphalocele patients available, many comparisons were made between much smaller groups due to the specificity of the clinical questions and the limited applicability of these variables in the cohort. For example, for clinical questions about the non-operative management for patients with giant omphalocele, only patients registered as having giant omphalocele that received non-operative management could be included for the analysis. Panel members were presented with an overview of raw, anonymous, uncorrected data. For each PICO question, they received a data sheet with incidences of outcomes for patient groups that had undergone the intervention of interest (e.g. vaginal delivery) or the comparison of interest (e.g. cesarian section). The specific interventions and comparisons of interest associated with each PICO question are listed in Table 1. To increase interpretability, for each clinical question, an overview of patient characteristics was provided. All data analyses to process EPSA data were performed using R (version 4.4.1). Panel members evaluated the perceived effectiveness of interventions using SOFs and a Likert-type scale, rating EPSA data on a spectrum from harmful to beneficial (see Fig. 2).

**Table 1:** Clinical questions and their match with registered datapoints in the EPSA

	Question	Intervention / Comparison of interest	Evaluated outcomes	Necessary variables	Available relevant variables	Selected as supplementary evidence
Module 1: Prenatal care	1. Should whole exome sequencing (WES) be offered to parents expecting a child with omphalocele after karyotyping(KT) or chromosomal microarray (CMA) has been performed?	Whole exome sequencing AND karyotyping or CMA / karyotyping or CMA alone	Diagnostic yield (additional diagnoses with WES that was not found with KT or CMA)	Karyotyping yes/no; if yes, diagnosis yes/no, CMA yes/no; if yes diagnosis yes/no yes/no, WES yes/no; if yes diagnosis yes/no.	DNA diagnostics yes/no, conclusion DNA diagnostics	No, data too unspecific *
	2. Should vaginal delivery or planned cesarean section be advised in mothers expecting a child with a non-giant omphalocele?	Vaginal Delivery / Planned Cesarean Section	Mortality, any proxies for morbidity	Type of omphalocele, type of delivery: vaginal or elective cesarean, death of patient yes/no, any proxies for morbidity	Type of omphalocele normal/giant, Liver herniation yes/no, Elective cesarean yes/no, Vaginal delivery yes/no, emergency cesarean yes/no, Date of death, use of oxygen yes/no, Ventilation yes/no; if yes duration of mechanical ventilation	Yes
	3. What is the prognostic value of prenatal measurements for neonatal outcome? The panel explored the following measurements: A. Liver herniation as observed on prenatal ultrasound B. Omphalocele ratio’s as observed on prenatal ultrasound C. Observed to expected lung volume as observed on prenatal MRI	-	Mortality, any proxies for morbidity, ability for primary closure of the omphalocele	A. Liver herniation prenatally detected, mortality rate, any proxies for morbidity, primary closure yes/no B. Circumference omphalocele, circumference abdomen, diameter omphalocele, death of patient yes/no, any proxies for morbidity, primary closure yes/no C. Prental MRI yes/no, Observed to expected total lung volume, death of patient, any proxies for morbidity, primary closure yes/no	A. Prenatal ultrasound yes/no, Prenatal suspicion of omphalocele diagnosis at … weeks, unknown or postpartum B. Diameter abdominal wall defect(cm) at 20 weeks gestation, abdomen diameter (cm), circumference omphalocele (cm) C. - Available outcome variables: Date of death, use of oxygen yes/no, Ventilation yes/no; if yes duration of mechanical ventilation, primary closure yes/no	No, too much missing data for prenatal measurements **
Module 2: Postnatal Care	1. Should enteral feeding in neonates with giant omphalocele that undergo staged closure of the abdominal wall, be introduced before the abdominal wall is closed?	Enteral feeding before closure of the abdominal wall/Enteral feeding after closure of the abdominal wall.	Operative complications, any proxies for morbidity or feeding outcomes	Type of omphalocele, Type of closure (staged), date start enteral feeding, date of surgery, no. operative complications, any proxies for morbidity or feeding outcomes	Type of omphalocele normal/giant, Liver herniation yes/no, Staged closure yes/no, date start enteral feeding, date of surgery, no. operative complications, date of surgery, date of discharge, date of start enteral feeding, date of full feeding, line sepsis yes/no	Yes
	2. What are the (un)desirable effects of spontaneous breathing versus intubation and mechanical ventilation in neonates with giant omphalocele that undergo staged closure of the abdominal wall?	Spontaneous breathing during staged repair / intubation and mechanical ventilation during staged repair	Operative complications, time to surgery, any proxies for morbidity or feeding outcomes, pain and/or discomfort of the neonate	Type of omphalocele, Type of closure (staged), ventilation yes/no, any proxies for morbidity or feeding outcomes, pain and/or discomfort of the neonate	Type of omphalocele normal/giant, Liver herniation yes/no, Staged closure yes/no, ventilation yes/no, days till surgery, length of postoperative stay (operation date – discharge date), time to full feeds (date of start enteral feeding – date of full feeding), line sepsis yes/no, respiratory tract infection yes/no	Yes
Module 3: Closure of the abdominal wall	1. Should non-operative management and delayed closure versus staged closure be preferred in patients with a giant omphalocele?	Non-operative management and delayed closure / staged reduction and surgical closure	Mortality, any proxies for morbidity or feeding outcomes duration of mechanical ventilation	Type of omphalocele, Type of closure (staged or non-operative with delayed closure), death of patient yes/no, Ventilation yes/no; if yes duration of mechanical, any proxies for morbidity or feeding outcomes	Type of omphalocele, Type of closure (primary, staged or delayed), days until surgery, ventilation yes/no; if yes, duration of ventilation, days till surgery, date of surgery, date of discharge, date of start enteral feeding, date of full feeding, line sepsis yes/no, no. operative complications	Yes
	2. What substance(s) should be used for non-operative management of a giant omphalocele?	Multi comparison between substance types	Time to epithelization, number of complications, toxicity	Type of omphalocele, Type of closure (non-operative with delayed closure), no. operative complications, toxic effects observed, date of start treatment, date of epithelization	Type of omphalocele normal/giant, Liver herniation yes/no, Type of closure: delayed, type of surface cover (dry, wound dressing, flammazine or other), days until surgery, operation date discharge date, date of start enteral feeding, date of full feeding	Yes
	3. Are there specific interventions for staged reduction in patients with giant omphalocele to be preferred over other available approaches?	Multi comparison between staged reduction methods	Mortality, Need for ventilation, time until surgery, duration of ventilation, any proxies for morbidity or feeding outcomes, operative complications	Type of omphalocele, Intervention for staged closure, time until surgery, ventilation yes/no; if yes duration of ventilation, no. operative complications, any proxies for morbidity or feeding outcomes	Type of omphalocele normal/giant, Liver herniation yes/no, Type of closure: staged, closure abdominal wall with silo, closure abdominal wall with patch, days until surgery, ventilation yes/no; if yes, duration of ventilation, days till surgery, date of surgery, date of discharge, date of start enteral feeding, date of full feeding, line sepsis yes/no, no. operative complications	No, interventions too unspecific. ***
	4. What is the optimal timing for surgery in patients with non-giant and giant omphalocele? A. Should primary closure of a non-giant omphalocele be done before or after the age of 2 days of life? B. Should delayed closure after non-operative management of patients with a non-giant omphalocele be done before or after the age of 1 year of life?	A. Primary surgical closure on day 0–2 of life / Primary surgical closure after day 2 B. Delayed closure (after initial non-operative management) before age 1 year old / Delayed closure (after initial non-operative management) after age 1 year old	A. Mortality, postoperative complications, any proxies for morbidity or feeding outcomes B. Postoperative complications, length of postoperative hospital stay	A. Type of omphalocele, days until surgery, postoperative complications, any proxies for morbidity or feeding outcomes B. No. postoperative complications, length of postoperative hospital stay.	A. Type of omphalocele normal/giant, Liver herniation yes/no, Type of closure: primary closure, date of birth, date of surgery, ventilation yes/no; if yes, duration of ventilation, days till surgery, date of surgery, date of discharge, date of start enteral feeding, date of full feeding, line sepsis yes/no, operative complications B. Type of omphalocele normal/giant, Liver herniation yes/no, Type of closure: delayed closure, date of birth, date of surgery, date of discharge	Yes

*The EPSA dataset didn’t specify the different types of diagnostic tests available for DNA testing. Therefore, we were unable to separate the patients that were diagnosed after whole exome sequencing from those who only had karyotyping or chromosomal microarrays

** While the OC/AC ratio and prenatal ultrasound findings such as liver herniation are collected through EPSA, the case completeness was very low

*** The EPSA dataset didn’t specify the different types of staged reduction. For options ‘patch’ and ‘silo’ it was unclear what type of intervention was provided (e.g. Surgical silo, non-surgical silo). For the option ‘suspension’ there was a lack of agreement between panel members as to the definition of suspension, this data was therefore excluded

An example of a completed SOF is available in the supplementary materials (S1b). The number of votes for each level of perceived effectiveness (Large benefit, small benefit, no effect, small harm, large harm) was presented to panel members during the evidence to decision phase; this was called the ‘evidence supplement’. During a two-day face-to-face meeting, recommendations for all questions were discussed based on the reviewed literature, evidence supplement and/or opinion of experts.

### Evaluation of REGARD

To evaluate REGARD we collected the number of clinical questions for which EPSA data was available (feasibility) and the number of recommendations to which the evidence supplements contributed (added value). Additionally, a survey was distributed amongst panel members using Survey Monkey© version 2024 to evaluate their experiences with this method (acceptability among panel members). The evaluation survey was sent to all 23 clinical experts on the panel. To prevent recall bias, experts were given a 6-week period to complete the survey in which 2 reminders were issued. The survey evaluated their experiences with the methodology as a whole (e.g. Combined use of Guidelines 2.0, GRADE ETD framework and REGARD), but also whether they thought REGARD was of additional value. Panel members voted on statements about the methodology on a five-point Likert scale ranging from completely disagree [1] to completely agree [5]. Last, panel members were asked to indicate how much time they spent on completing the SOFs. Ethical approval and consent to participate were considered not applicable in this study because no patient data or sensitive personal information were collected. The survey was conducted among clinical experts who had already participated in the guideline development panel in their professional capacity. Participation in the survey was voluntary, participants were aware their answers could be used in a scientific publication, and responses reflected professional experiences with the methodology rather than personal or health-related information. As such, the study did not fall under the scope of human subject research requiring formal ethics approval.

## Results

### Feasibility

The prioritization exercise led to the selection of twelve clinical questions covering areas related to prenatal care, postnatal care and closure of the abdominal wall. After matching the required EPSA variables for analysis to the specific clinical questions, seven questions showed an adequate match, and five questions showed an inadequate match (see Table 1). An inadequate match was either due to excessive missing data for the required variables (1 question), necessary datapoints not being included in the EPSA (2 questions), or there being a lack of specificity of the registered intervention (2 questions).

Panel members used REGARD for all seven questions with an adequate match. The evidence supplement initiated a discussion on why panel members perceived a certain effect in the presented data, whether certain patient characteristics may have acted as confounding factors, and what the panel members’ interpretation of the data could imply for a potential recommendation.

### Added value

**Fig. 3:**
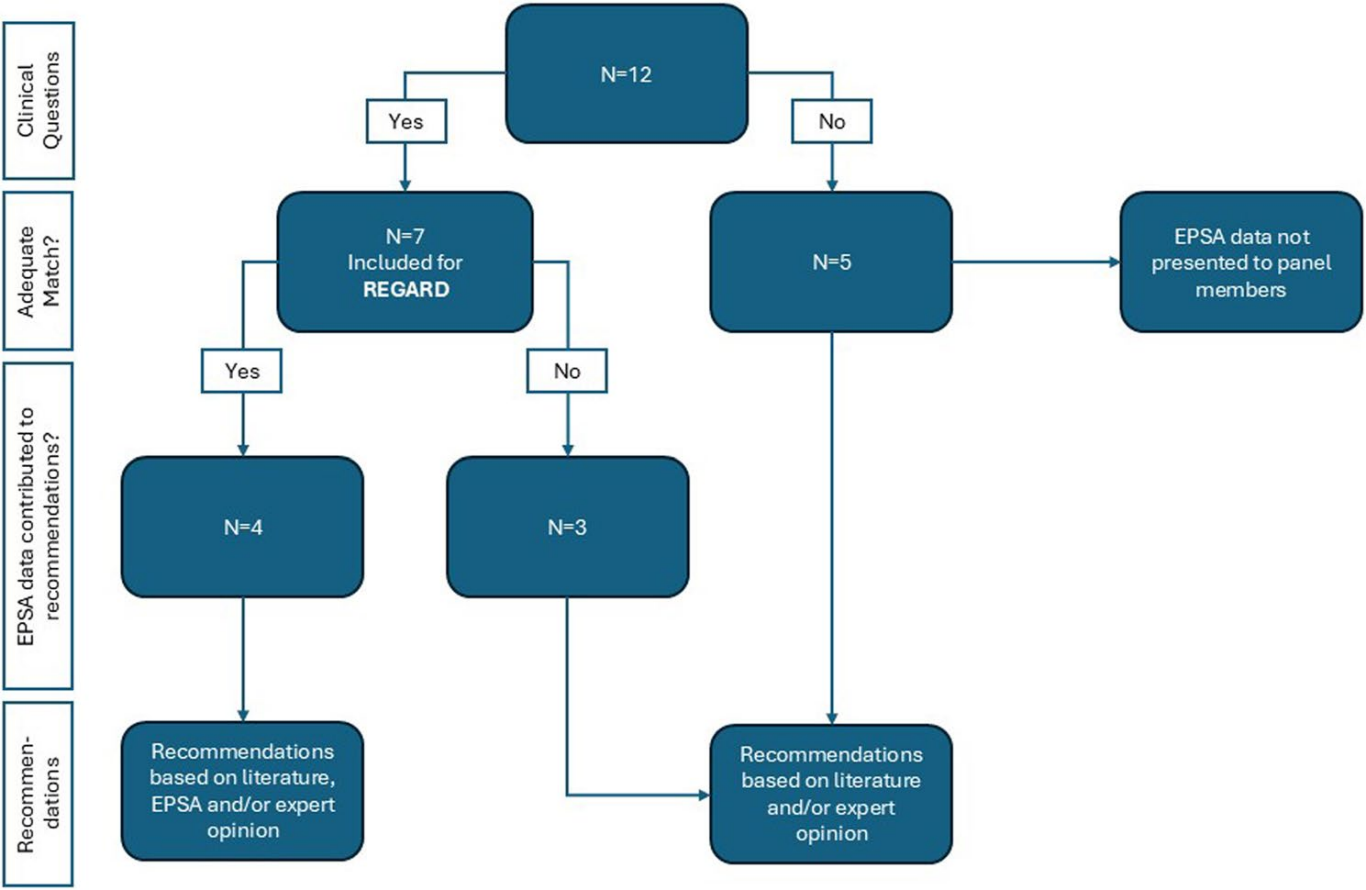
Schematic overview of use of EPSA data in the development of the Omphalocele guideline

In four questions, the evidence supplement contributed to the panel members’ decision on a recommendation. The data contributed to deciding on recommendations, as panel members either felt it strengthened their confidence in a potential positive effect (2 questions), or increased their certainty that a particular intervention did not lead to any harmful effects (2 questions). The main reasons for not considering the evidence supplement in the development of a recommendation were: uncertainty about the validity of the observations due to small sample sizes (3 questions), and concerns about the association between the observed outcomes and the intervention. Panel members mainly had concerns about the association between the observed outcomes and the intervention in case the registry data lacked information on the clinical indication for a chosen treatment and therefore limited the ability to draw unbiased conclusions about treatment effectiveness (2 questions). Figure 3 provides a schematic summary of the use of the EPSA data during the guideline development.

### Acceptability

Acceptability of REGARD by panel members was evaluated via a digital survey. The outcomes of the evaluation survey are listed in Table 2. At the end of the 6-week period the survey was completed by 22 experts.

**Table 2:** Panel member experiences with the methodology

Statement	% Agree or completely agree	Weighted average 5-point Likert scale
**Statements about the methodology as a whole**		
To my experience, the applied methodology provided enough structure	88.9%	4.17
To my experience, the applied methodology contributes to a higher quality guideline	83.3%	4.06
To my experience, the applied methodology contributes to increased validity of the final product	83.3%	4.00
I will likely participate in another guideline project where similar methodological approaches are applied	100%	4.61
**Statements specifically about the use of EPSA data (REGARD)**		
Overall, the evaluation of EPSA data through structured observation forms was a valuable addition to the guideline process	77.8%	3.94
The EPSA data and summary of structured observation forms contributed to constructive discussions during the evidence to decision phase	92.3%	4.31
To my opinion, the structured observation forms and evaluation of EPSA data could have more added value in the future, if the number of registered patients is growing and data collection is further improved	94.4%	4.56

**Time investment**	**Minutes spend (median)**	**Interquartile range (50%)**
How much time (specified in minutes) did you spend completing the structured observation forms? This includes studying the presented EPSA data	60 min	52.5–153.8 min

## Discussion

In this study we aimed to evaluate REGARD, an innovative methodology designed to strengthen the guideline development process for rare diseases. For four clinical questions, the supplementary EPSA evidence contributed to panel members’ decision on a recommendation, either by strengthening the panels’ confidence that there is a potential positive effect, or by increasing their confidence that a particular intervention does not result in any harmful effects. By using REGARD, the number of questions for which the panel had to solely rely on expert opinion decreased. An adequate match between EPSA variables and information necessary for the PICO questions was found for seven out of twelve PICOs. This contributed to the development of an evidence-based guideline and formed a first attempt within ERNICA to connect registry data to guideline development as part of a wider cyclical approach to quality improvement [2].

We realize there are limitations in the link between EPSA and the omphalocele guideline, as the EPSA is a clinical audit and the database is not a priori developed to answer the clinical questions in our guideline. However, the main goal of using registry data was not to conduct a tailored study to answer the guideline questions. REGARD is specifically designed for the context of rare diseases, where published (high-quality) evidence is scarce or absent and registry data may provide valuable insights. By integrating registry data with REGARD, guideline panels have something to start their discussion, rather than relying solely on expert opinion. The use of REGARD should therefore be understood as an effort to incorporate the best available evidence.

To optimize the use of REGARD in future guidelines, harmonized data collection is essential. If the collected data can be aligned with clinical issues prioritized by expert clinicians, EPSA data can support the analysis of practice variation, both across centers and between current practices and the ‘desired quality of care standard’. Combined with exploration of the underlying reasons for treatment decisions and the registration of patient characteristics that allow for case mix correction, this would enable a more informed assessment of whether such variation is undesirable and warrants guideline revisions. An important barrier that we encountered was the lack of clear definitions for some datapoints, which impeded optimal use of the data. However, the involvement of clinical experts in interpreting the definitions allowed us to utilize the majority of the data. The lack of clear definitions is not merely a limitation of the dataset but reflects a broader issue of definition heterogeneity evident in literature on omphalocele, as well as other rare diseases [16, 17]. Moving forward, data points must be clearly and consistently defined to leave no room for interpretation by the person registering. This need has been recognized in the field of pediatric surgical research, which explains the growing development of Core Indicator Sets (CIS) and Core Outcome Sets (COS) [18–23]. The COMET initiative was introduced to collate and stimulate relevant resources, both applied and methodological, to facilitate the exchange of ideas and information, and to foster methodological research in this area [24].

Despite the clinical questions and EPSA variables not being a perfect match, a total of 86 out of 98 variables (88%) of EPSA omphalocele variables were used in the analyses performed. Due to the 10 year data registration period, namely from 2013 till 2023, the number of 251 included omphalocele patients is relatively high when comparing it to published cohort studies, which often include small numbers of patients. However, for some clinical questions, the patient groups were small due to the specificity of the clinical questions, with fewer than ten patients per group for comparison. Given these small sample sizes, we chose not to apply statistical corrections and instead opted to provide an overview of patient characteristics and outcomes, leaving interpretation to the clinical experts. Although we provided extensive guidance and information on how to complete the structured observation forms based on the data and how to rate perceived effectiveness, some of our panel members reported they were missing the statistical information and expressed this would have helped them to better interpret the data.

Differences in patient characteristics between intervention groups, arising from the use of real-world data, may be seen as a limitation, but it also offers significant benefits. Due to the rarity and severity of many congenital anomalies, there is a need for patient tailored treatment [25, 26]. Conducting randomized controlled trials is therefore extremely challenging, resulting in long inclusion periods and low patient numbers, often due to strict inclusion criteria. To reduce reliance on expert opinion alone in guideline development, observational studies with long-term outcomes and multicenter data are crucial. Our study also demonstrates the value of structuring expert opinion through tools like structured observation forms (SOFs), which enable the systematic documentation of clinical decisions. Incorporating international, multicenter, real-world registry data further supports this by capturing comprehensive patient information. While statistical correction is ideal, our pilot showed that even uncorrected data analyses can offer valuable insights for guideline development.

This pilot with REGARD is a successful example of close collaboration between the EPSA project manager, guideline methodologist and our multidisciplinary guideline panel. As the diseases ERNICA covers are rare, so are the experts that we can involve for projects like this. Therefore, we evaluated REGARD with them, asking for their opinion about the process and added value of this way of using EPSA data as supplementary evidence. The majority of panel members reported that they considered the REGARD methodology of additional value to the guideline process. Additionally, 92,3% of the participants in the guideline development meeting agreed that the supplementary data from EPSA contributed to the quality of the panel discussions. It is likely that this is due to the fact that the data created an incentive for critical evaluation of several aspects of the issues discussed. When asked about the potential of the methodology for ongoing guideline development, 94,4% of panel members agreed that REGARD could have greater value in the future.

Not only the amount of available data, but also the data quality should be considered in the future. Data quality verification was conducted on the Dutch branch of the EPSA registry for the years 2018 to 2021. The quality assessment was done by peer visitation and included the evaluation of case completeness and data accuracy. Case completeness was evaluated using financial reimbursement data. Accuracy was assessed in a 10% sample by reviewing the source data –electronic patient records – and determining the agreement between the original records and EPSA data [27]. The assessment revealed a case completeness of 82% and summated data accuracy of 77% for omphalocele [27]. The discrepancies observed in data accuracy were attributed to the lack of comprehensive definitions and the use of conditional variables. Additionally, the verification was performed on the Dutch data, while our research was conducted on the entire EPSA population, including international data. However, awareness of these data pitfalls enabled us to interpret the data in a more nuanced manner. Given the importance of data accuracy for successful clinical audit implementation, addressing the issues related to definitions is essential to expand the number of patients in the dataset and advance the integration of EPSA data into quality improvement initiatives [28]. Moving forward, the EPSA team proceeds to focus on international data validation and the development of targeted improvement plans.

Finally, an important aspect to consider is the cost and time investment of experts for the development of this rare disease guideline. A significant amount of time was invested in this innovative methodology due to the analysis of a relatively large number of research questions, interpretation of data by clinical experts, and the summarization of completed structured observation forms. Panel members reported spending anywhere between 30 and 600 min (median 60 min, IQR 52.5-153.8) to interpret the EPSA data, on top of the time they spent studying the literature summaries and attending the guideline panel meetings. Given the limited pool of experts available for guideline development projects, all of whom also carry significant clinical responsibilities, further refinement of the method to reduce the time burden of data interpretation is warranted. Nevertheless, all panel members reported that they would participate in a future project using a similar methodology.

This study represents an important step toward achieving cyclical quality improvement and more robust guideline development rare diseases. With the introduction of REGARD we demonstrated how real-world data can be systematically integrated into guideline development, offering a valuable complement to traditional forms of evidence. Despite challenges such as limited alignment between available data and clinical questions and small sample sizes, our study shows that registry-based evidence can meaningfully strengthen recommendations and reduce reliance on expert opinion alone. Beyond rare diseases, this approach may also be valuable in guideline development for conditions where randomized controlled trials or other high-quality studies are scarce. In such contexts, real-world data can provide essential insights into patient characteristics, interventions, and outcomes that would otherwise remain unavailable. The introduction of REGARD illustrates how structured data collection can enhance the relevance of (rare disease) registries, potentially stimulating registry participation across diverse clinical areas.

## Conclusion

This study evaluates an innovative approach to evidence-based guideline development for rare diseases, by using real-world data as an evidence supplement as part of an integrative quality improvement system. While the methodology requires refinement and the dataset needs optimization, we believe REGARD holds significant promise as a tool for the development of guidelines for rare diseases. Future research should focus on the harmonization of data collection and the establishment of clear, standardized definitions for registry variables to reduce interpretation bias and improve data usability. Expanding multicenter collaboration and registry implementation to increase sample sizes will be essential to enable statistical correction and more robust outcome analysis, which will allow for refinement of the tested methodology.

## Supplementary Information

Supplementary Material 1.

## Data Availability

Anonymized survey data supporting the findings of the panel member evaluation are available from the corresponding author upon reasonable request. Other results can be validated with the Evidence to Decision tables from the ERNICA guideline on omphalocele (Irvine et al., 2025; ERNICA Evidence Based Guideline on Omphalocele. Submitted for publication to Orphanet J Rare Dis.). Due to restrictions on the use of EPSA data, the data presented to the panel members for REGARD, is not available.

## Abbreviations

EPSA: European Pediatric Surgery Audit
ERNICA: European Reference Network for Rare Inherited and Congenital Anomalies
REGARD: Real-world Evidence for Guidelines Addressing Rare Diseases
SOF: Structured Observation Form
PICO: Patient population, Intervention, Comparison, Outcome
