# Supplementary material for: Enhancing rare disease guideline development with real-world data: a method evaluation
Source: BMC Med Res Methodol. 2026 Feb 14;26:61. doi: 10.1186/s12874-026-02802-7 (PMC13011638; doi:10.1186/s12874-026-02802-7)
Supplement: Supplementary file 1 — Supplementary Material 1. [file 12874_2026_2802_MOESM1_ESM.docx]

# Supplement 1. Methods

1. **Aim and target audience guideline**

The aim of this guideline is to provide recommendations on various multidisciplinary aspects of care for omphalocele patients and their families. The guideline is targeted to all European health care providers that are involved in the care for omphalocele patients both pre- and postnatally. This guideline is not specifically targeted to, but can be informative for, families and caregivers of patients.

1. **Validity of the guideline**

This guideline is valid from the moment of publication, until the publication of its update. In general, a guideline will undergo evaluation every 5 years. Consequently, the board of the ERNICA will install a new workingroup in 2030 to evaluate which modules are need of an update and if there is a need to develop new modules based on the latest insights. ERNICA is primarily responsible for the validity of the guideline and timely revision. The cooperating expert centres for omphalocele share the responsibility to inform ERNICA if new developments in research or clinical practise raise the need for evaluation before the 5 year mark.

1. **Patient population**

This guideline covers the care for omphalocele patients from prenatal diagnosis until postnatal intervention to close the abdominal wall. Long term consequences and cosmetic interventions are not included as topics of interest in this guideline.

According to the Orphanet, Snomed, ICD10 and ICD11 coding systems, this considers:

ORPHA:  660, 695032, 695038

SNOMED: 64176007

ICD 10: Q79.2

ICD-11: LB01

1. **Guideline development group**

A multidisciplinary guideline development panel (GDP) including 5 maternal and fetal medicine specialists, 6 neonatologists, 12 pediatric surgeons, a methodologist, an EPSA project manager and an implementation coordinator was selected from all ERNICA hospitals that are accredited as expert centres for omphalocele. As omphalocele is such a rare disease and no formal patient support organizations exists, we failed to identify patient representatives willing to be part of the guideline development panel. Instead, two patient representatives that were suggested by participating clinicians contributed to the development process by giving written input at various stages throughout the development process. The guideline development group consisted in total of 26 experts from 12 European countries.

Table A. The guideline development group

| Professional |  | Affiliation and country | GDG position |
| --- | --- | --- | --- |
| Carmen Mesas Burgos, MD, PhD, FEBPS | Consultant Paediatric Surgeon, Associate Professor | Karolinska University Hospital, Stockholm – Sweden | Chair |
| Prof. Alexandra Benachi, MD, PhD | Maternal and Fetal Medicine specialist | Assistance Publique-Hopitaux de Paris. Hopital Antoine Béclère. Université Paris Saclay. CRMR Hernie de Coupole Diaphragmatique. Clamart. France | Chair |
| Willemijn Irvine, MSc | Guideline Methodologist | Qualicura, Breda  Erasmus MC Sophia Childrens Hospital, Rotterdam – the Netherlands | Methodologist |
| Simon Eaton | Professor of pediatric surgery, nutrition and metabolism | UCL, London – United Kingdom | Panel expert |
| Marc Miserez | Abdominal Surgeon | Leuven UZ – Belgium | Panel expert |
| Julia Brendel | Pediatric Surgeon | Hannover Medical School – Germany | Panel expert |
| Katrin Zahn | Pediatric Surgeon | Universitätsmedizin Mannheim – Germany | Panel expert |
| Lucas Matthyssens | Pediatric Surgeon | UZ Gent – Belgium | Penel expert |
| Alberto Sgrò | Pediatric Surgeon | University of Padua – Italy | Panel expert |
| Rony Sfeir | Pediatric surgeon | CHRU, Lille – France | Panel expert |
| Elisabet Gustafsson | Pediatric Surgeon | Akademiska Sjukhuset Uppsala - Sweden | Panel expert |
| Henrik Røkkum | Pediatric Surgeon | Oslo University Hospital – Norway | Panel expert |
| Lucia Migliazza | Pediatric Surgeon | ASST Papa Giovanni XXIII di Bergamo – Italy | Panel expert |
| René Wijnen | Pediatric Surgeon | Erasmus MC Sophia Childrens Hospital, Rotterdam – the Netherlands | Panel expert |
| Annika Mutanen | Pediatric Surgeon | Helsinki Children's Hospital – Finland | Panel expert |
| Udo Rolle | Pediatric Surgeon | Universitätsklinikum Frankfurt – Germany | Panel expert |
| Anne Dariel | Pediatric Surgeon | CHU de Marseille - Hopital de la Timone – France | Panel expert |
| Ausra Lukosiute-Urboniene | Pediatric Surgeon | Hospital of Lithuanian University of Health Sciences Kauno Klinikos | Panel expert |
| Alexandre Vivanti | Maternal and Fetal Medicine specialist | GHU Paris-Sud - Hôpital Antoine Béclère | Panel expert |
| Nina Peters | Maternal and Fetal Medicine specialist | Erasmus Medical Center, Rotterdam – The Netherlands | Panel expert |
| Peter Conner | Maternal and Fetal Medicine specialist | Karolinska University Hospital | Panel expert |
| Eglė Machtejevienė | Maternal and Fetal Medicine specialist | Lithuanian University of Health Sciences– Lithiuania | Panel expert |
| Francesca Russo | Maternal and Fetal Medicine specialist | UZ Leuven – Belgium | Panel expert |
| Ana Sanchez Torres | Neonatologist | Hospital Universitario La Paz, Madrid – Spain | Panel expert |
| Alena Kokesova | Neonatologist | University Hospital Motol, Prague, Czech republic | Panel expert |
| Hans Jorgen Stensvold | Neonatologist | Oslo University Hospital | Panel expert |
| Florian Kipfmueller | Neonatologist | Universitatsklinikum Bonn | Panel expert |
| Sofia Apostolidou | Neonatologist | Universitätsklinikum Hamburg-Eppendorf | Panel expert |
| Mohamed Riadh Boukhris | Neonatologist | CHRU Lille – France | Panel expert |
| Iris den Uijl | ERNICA Project manager | Erasmus MC Sophia Childrens Hospital, Rotterdam – the Netherlands | Project support and management |
| Olivia Spivack | Implementation coordinator | Erasmus MC Sophia Childrens Hospital, Rotterdam – the Netherlands | Panel expert on implementation |
| Linde Margriet van der Kamp | EPSA project manager | Erasmus MC Sophia Childrens Hospital, Rotterdam – the Netherlands and Dutch Institute for Clinical Auditing | EPSA data specialist |

Table 1: Guideline development group members and their affiliations.

All members of the steering group declared their interests. None of the GDG members had conflicting interests for this guideline. Completed declaration of interest forms of all GDG members are available on request at the ERNICA office.

1. **Implementation**

The implementation of the guideline and the practical feasibility of the recommendations were taken into account during the different phases of guideline development. In doing so, explicit consideration was given to factors that could promote or hinder the implementation of the guideline in practice. Specific barriers and facilitators to applicability of the recommendations are included in the evidence to decision frameworks (EtD) if relevant.

1. **Analysis of clinical care gaps**

During the initiation phase,  analysis, prioritization and listing of clinical gaps was done through email discussions and google forms and later refined during online and in-person meetings.  The highest ranking questions were selected to be part of the guideline.

1. **Questions and outcomes**

The analysis of clinical care gaps formed the basis for the questions for the guideline. The GDP chose to address prenatal as well as postnatal care topics. The GDG members pre-selected outcomes of interest to reflect the main concerns in omphalocele care and as we knew beforehand the evidence would be scarce, surrogate outcomes that could be allowed were discussed for all questions. All questions were refined and structured according to the PICO framework.

1. **Literature search and selection of literature**

A systematic literature search was conducted by a professional biomedical information specialist in the Erasmus Medical Center. The search was conducted in Medline (all), EMBASE and Cochrane. The full search strategy is available in supplement S4. The methodologist and chairs double blinded screened all results (n =808) based on title and abstract and excluded the following:

- cross-sectional studies
- animal studies
- in vitro studies
- Original studies with <5 included patients
- Articles published before 2000 (if there were no publications found published after 2000, this criterion was dropped)
- Case reports
- Expert opinion
- Letters to the editor
- Editorials
- Reviews

Eligibility was assessed based on a match with one or more PICO questions and their outcomes of interest.

1. **Pilot intervention**

In this guideline we piloted the use REGARD (**R**eal-world **E**vidence for **G**uidelines **A**ddressing **R**are **D**iseases ). With REGARD registry data is integrated in guideline development by using structured observation forms (SOFs). A SOF is a matrix that experts use to rate their perceived effectiveness of an intervention on selected outcomes on a spectrum from harmful to beneficial using a Likert type scale, see figure 1. Available EPSA data variables were matched to the clinical questions to be able to present data relevant to the clinical questions. At the time of data extraction, the EPSA database contained data on 251 omphalocele patients included from 2013 till 2023. However, comparisons were made between much smaller groups due to the specificity of the clinical questions. As a result, we presented panel members with an overview of raw, uncorrected data. For each clinical question, an overview of patient characteristics and outcomes was provided for each intervention group. Based on the method suggested by Pai et al. [1] experts rated their perceived effectiveness based on the presented EPSA data on a spectrum from harmful to beneficial on a Likert type scale, see figure 1.

Figure 1. Structured observation of cases


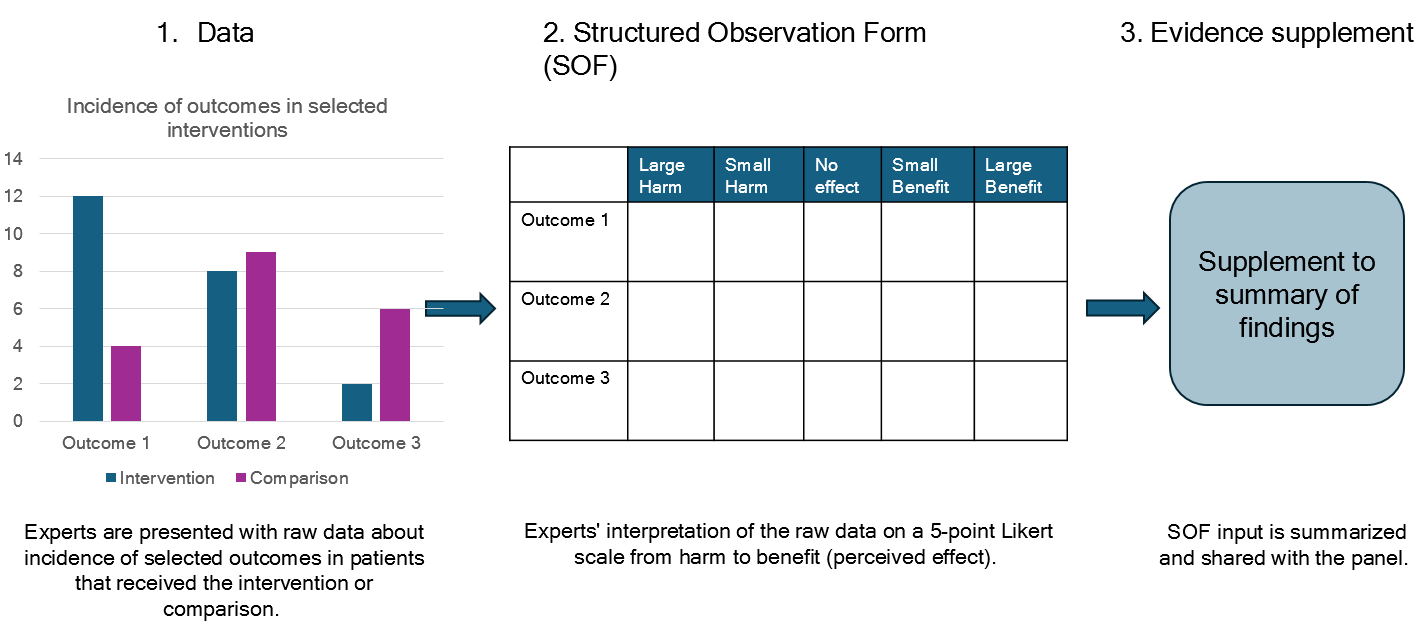


 A summary of the experts’ observations was provided next to a summary of literature (if available) during the ‘evidence to decision’-phase. An example of a completed SOF is available at the end of this supplement (S1b).

1. **Summary of findings**

Data extraction and bias assessment was performed by the and reviewed by a panel  expert. Any disagreement between reviewers was resolved by either discussion or by consulting a third reviewer if necessary. Based on the ‘Grading Recommendations Assessment, Development and Evaluation’ (GRADE) methodology [2] , all of the evidence on one outcome of interest, was collectively assessed as a ‘body of knowledge’ to determine the quality of evidence for that outcome. The quality of the body of knowledge was assessed following the GRADE methodology. GRADE results in the categorization of evidence into four levels: high, moderate, low and very low. These levels can be interpreted according to Table B.

Table B. Grading of Recommendations, Assessment, Development, and Evaluations

| GRADE | Definition |
| --- | --- |
| High | We are very confident that the true effect lies close to that of the estimate of the effect. |
| Moderate | We are moderately confident in the effect estimate: The true effect is likely to be close to the estimate of the effect, but there is a possibility that it is substantially different |
| Low | We are limitedly confident in the effect of estimate: The true effect may be substantially different from the estimate of the effect. |
| Very Low | We have very little confidence in the effect estimate: The true effect is likely to be substantially different from the estimate of effect |

All summary of findings tables are in the supplementary material (S2)

1. **Considerations and recommendations**

To determine the strength and direction of a recommendation, the Evidence to Decision(EtD) framework including ‘panel voice’ was used . The purpose of EtD frameworks is to help people use evidence in a structured and transparent way to inform decisions in the context of clinical recommendations and other health system or public health decisions [3].  The EtD framework was used as a guide to structure a two days consensus meeting in Stockholm, Sweden (November 2024) with most panel members present. To prepare for this meeting, a summary of findings of all modules was distributed among panel members, accompanied by an electronic survey covering all the aspects of the evidence to decision framework (panel voice). This survey prepared panel members for the way of thinking that is necessary to discuss recommendations according to the evidence to decision framework and highlighted important discussion point upfront. Panel members that could not participate in the meeting, also received this survey so that their insights could be included in the discussion. During the consensus meeting, considerations, recommendations and research needs following the literature analysis and panel voice survey were discussed. A summary of the completed SOFs was presented as an evidence supplement and discussed alongside the summary of findings based on published literature. During this two day consensus meeting the evidence to decision framework led the GDP to a collective agreement on the type of recommendation for all PICO questions. The GDP decided between five recommendation options and chose uniform wording of recommendations for each of them as displayed in Table C. The exact wording of the recommendations was later agreed upon through email and electronic voting.

Table C. Types of recommendations and their wording

| Type of recommendation | Wording |
| --- | --- |
| **Strong recommendations against the intervention** | The panel recommends against / to refrain from |
| **Conditional/weak recommendation against the intervention** | The panel suggests against / to refrain from |
| **Conditional/weak recommendation for either the intervention or the comparison** | The panel suggests either … or … |
| **Conditional/weak recommendation for the intervention** | The panel suggests …. |
| **Strong recommendation for the intervention** | The panel recommends …. |

In accordance with the GRADE method, a low probative value of conclusions in the systematic literature analysis does not exclude a strong recommendation in advance, and weak recommendations are also possible with a high probative value. The strength of the recommendation is always determined by weighing all relevant arguments.

In addition to evidence-based recommendations, this guideline includes Good Practice Statements (GPS). These are recommendations that are considered essential for clinical practice but are not directly supported by a systematic review of the evidence. GPS are typically based on expert consensus, ethical considerations, or widely accepted standards of care.

All main outcomes and considerations for each question are summarized in the modules, full EtD tables are available in the supplementary materials (S3).

1. **Evaluation and authorization**

A draft of the guideline was submitted to all expert centres and patient representatives involved in the care for patients with omphalocele and affiliated with ERNICA. Comments from this peer review were collectively discussed within the GDP, resulting in adaptations and refinements of the guideline. Experts from non-omphalocele centres were also invited for the commentary round, as well as international specialists not associated with ERNICA. All reviewer comments were summarized and reviewers received a rebuttal that included either confirmation their comments were included in the revised guideline or a motivation for not including their suggestion. During the authorisation phase the guideline was approved by all ERNICA registered omphalocele expert centres.

1. **Implementation &monitoring**

A central implementation support team (the ERNICA CIST) has been initiated within ERNICA. This team is dedicated to supporting the successful implementation of ERNICA’s guideline recommendations in clinical practice. An upcoming qualitative study will further explore the factors foreseen to hinder and/or facilitate implementation of several of theomphalocele guideline recommendations. This will be done with a view to selecting appropriate implementation strategies. We intend to employ the European Pediatric Surgical Audit (EPSA) as a mechanism to monitor guideline implementation. Data points aligning with the recommendations are therefore to be included in the EPSA omphalocele data set. This will allow us to monitor levels of guideline adherence, alongside other markers of care quality. This guideline will be assessed for possible revision in 5 years time (2030). If possible, we intend to use EPSA data to inform the need for/scope of revision

[1] Pai M, Yeung CHT, Akl EA, Darzi A, Hillis C, Legault K, et al. Strategies for eliciting and synthesizing evidence for guidelines in rare diseases. BMC Med Res Methodol 2019;19(1):67.

[2] Guyatt GH, Oxman AD, Vist GE, Kunz R, Falck-Ytter Y, Alonso-Coello P, Schünemann HJ. GRADE: an emerging consensus on rating quality of evidence and strength of recommendations. Bmj 2008;336(7650):924-6.

[3] Moberg J, Oxman AD, Rosenbaum S, Schünemann HJ, Guyatt G, Flottorp S, et al. The GRADE Evidence to Decision (EtD) framework for health system and public health decisions. Health research policy and systems 2018;16:1-15.

# Supplement 1b

## Structured observation form

PICO 2: Should babies with a small omphalocele be delivered vaginally or via elective cesarean section?

P= Omphalocele

I = Vaginal delivery

C= Elective cesarean section

O= Mortality, Morbidity

EPSA input

1. Based on the presented data from the EPSA, what is your perceiving of the effect* of ***vaginal delivery*** on:

|  | Large benefit | Small benefit | No effect | Small harm | Large harm | Missing information / Don’t know |
| --- | --- | --- | --- | --- | --- | --- |
| Mortality |  | x |  |  |  |  |
| Duration of mechanical ventilation |  | x |  |  |  |  |
| Time to first feeds |  |  | x |  |  |  |
| Time to full feeds |  | x |  |  |  |  |

1. To your perceiving, are the expected benefits/harms substantially different for different subgroups/for patients with specific characteristics?

The data does not suggest that vaginal delivery is generally considered to have a significant advantage. Since patients with the liver in the omphalocele are more often delivered by elective cesarean section, this implies that clinicians have likely used this as the basis for decisions regarding the mode of delivery. Therefore, there is a certain difference in terms of patient characteristics. However, the data shows that vaginal delivery works excellently for a large group of patients and can therefore often be recommended.

1. Any other remarks or important considerations?

No
